# The Dangers of Being a Small, Oligotrophic and Light Demanding Freshwater Plant across a Spatial and Historical Eutrophication Gradient in Southern Scandinavia

**DOI:** 10.3389/fpls.2018.00066

**Published:** 2018-02-02

**Authors:** Kaj Sand-Jensen, Hans Henrik Bruun, Tora Finderup Nielsen, Ditte M. Christiansen, Per Hartvig, Jens C. Schou, Lars Baastrup-Spohr

**Affiliations:** ^1^Freshwater Biological Section, Department of Biology, Faculty of Natural and Life Sciences, University of Copenhagen, Copenhagen, Denmark; ^2^Section of Ecology and Evolution, Department of Biology, University of Copenhagen, Copenhagen, Denmark; ^3^Natural History Museum of Denmark, University of Copenhagen, Copenhagen, Denmark; ^4^BioPix, Hobro, Denmark

**Keywords:** aquatic plants, acidification, biodiversity, environmental change, eutrophication, historical changes, Southern Scandinavia, species traits

## Abstract

European freshwater habitats have experienced a severe loss of plant diversity, regionally and locally, over the last century or more. One important and well-established driver of change is eutrophication, which has increased with rising population density and agricultural intensification. However, reduced disturbance of lake margins may have played an additional key role. The geographical variation in water chemistry, which has set the scene for – and interacted with – anthropogenic impact, is much less well understood. We took advantage of some recently completed regional plant distribution surveys, relying on hundreds of skilled citizen scientists, and analyzed the hydrophyte richness to environment relations in five contiguous South-Scandinavian regions. For three of the regions, we also assessed changes to the freshwater flora over the latest 50–80 years. We found a considerable variation in background total phosphorus concentrations and alkalinity, both within and between regions. The prevalence of functional groups differed between regions in accordance with the environmental conditions and the species’ tolerance to turbid waters. Similarly, the historical changes within regions followed the same trend in correspondence to the altered environmental conditions over time. Small submerged species decreased relative to tall submerged and floating-leaved species along the regional and historical eutrophication gradients. These changes were accompanied by systematically greater relative abundance of species of higher phosphorus prevalence. We conclude that species traits in close correspondence with anthropogenic impacts are the main determinants of local, regional and historical changes of species distribution and occupancy, while pure biogeography plays a minor role. Conservation measures, such as re-oligotrophication and re-established disturbance regimes through grazing and water level fluctuations, may help reduce the tall reed vegetation, restore the former richness of the freshwater flora and safeguard red-listed species, although extended time delays are anticipated in nutrient-rich regions, in which species only survive at minute abundance in isolated refugia.

## Introduction

The species richness of freshwater vascular plants has declined, both regionally and locally, throughout Europe during the last century. The change has been driven by loss and deterioration of habitats (Sand-Jensen et al., 2000; Körner, 2002; Sundberg, 2014). Many lakes have been reclaimed for agricultural use (Hansen, 2008). Similarly, streams have been channelized, deepened and exposed to dredging of surface sediments and plant cutting in order to enhance drainage effect and enable conversion of wet river meadows to dry arable land (Baattrup-Pedersen et al., 2003; Fehér et al., 2012). This profound stream regulation has caused the loss of all but the few submerged plant species able to cope with severe and frequent disturbance (Riis and Sand-Jensen, 2001). Reduced water level fluctuations in lakes and cessation of cattle grazing and mowing on lake banks probably have led to replacement of the species-rich, low-growing amphibian vegetation with a species-poor vegetation of tall emergent plants (Tyler and Olsson, 1997; Maad et al., 2009).

Increased application of fertilizers in agriculture and increased outlet of sewage from growing human populations during the 20th century led to turbid lake waters and alteration of the submerged vegetation from oligotrophic, bottom-dwelling species toward eutrophic canopy-forming or floating-leaved species tolerant of turbid waters (Hilt et al., 2013; Murphy et al., 2017; Sand-Jensen et al., 2017). Cessation of reed cutting, mowing and grazing of domestic livestock (Swedish Board of Agriculture, 2011; Hartvig, 2015) has contributed to change the open lake banks dominated by small competitively inferior plant species to tall, denser vegetation of emergent reed plants and bushes according to historical photographs and personal reports, though scientific analyses of the different impacts are lacking. Finally, deterioration of water quality for freshwater plants have involved acidification of softwaters during 1960–1990, followed by brownification by release of terrestrial humic substances when sulfur-emission from power plants and, thus, deposition of acids declined after 1990 (Stoddard et al., 1999; Roulet and Moore, 2006; Monteith et al., 2007; Ekström et al., 2011; European Environment Agency, 2014). Overall, eutrophication and land use changes in densely populated and cultivated lowland regions and, in parallel, acidification and brownification in sparsely populated regions with carbonate-poor soils, have contributed to changed richness and relative abundance of freshwater species of different life-form and trophic preference throughout Europe.

Though these fundamental changes of the freshwater flora have been reported from many local biotopes, the broad-scale relationships of freshwater species abundance to species traits and environmental conditions across large geographical regions have rarely been assessed. Here, we perform an analysis of richness-environment relationships in five contiguous lowland regions, two in Denmark (East-Denmark and West-Denmark) and three in Southern Sweden (Scania, Blekinge, and Småland). The five regions share almost the same species pool of about 90 species of aquatic vascular plants, but environmental conditions follow a pronounced gradient of decreasing nutrient levels and alkalinity (Alk) along with sparser human population density, proportion of agricultural land use and proportion of calcareous and clayish soils in the order: East-Denmark, West-Denmark, Scania, Blekinge, and Småland.

Historically, Denmark and Southern Sweden have had a species-rich and luxuriant flora of freshwater plants because of the shallow lowland streams and the many ponds and lakes of highly variable size and water chemistry (Schou et al., 2017). Freshwaters in Denmark and Scania, with their variable geology from clayish to sandy soils, large areas of high population density and intensive agriculture, but also scattered natural areas with low population density, may range from oligotrophy to hyper-eutrophy and from softwaters of low Alk to hardwaters of high Alk. Freshwaters in Blekinge and Småland, in contrast, may range from supra-oligotrophy to meso-eutrophy and from softwaters to medium hardwaters because of lower population density and agricultural activity, but more extensive forests on sandy soils overlying silicious bedrock of low weathering intensity (Andersson and Willen, 1999; Sjörs, 1999). The geographical gradient from Småland to Scania and Denmark may resemble the temporal changes of increasing nutrient richness taken place during the last 100 years most notably in the densely populated and intensely cultivated regions of Denmark and Scania. Moreover, if abundance of plant species changes in correspondence with their biological traits, as reflected by life-form, canopy height and nutrient preference (Grime, 1977; Sundberg, 2014; Baastrup-Spohr et al., 2017), we may expect a parallel change in the abundance of species with different biological traits across environmental gradients in space and time.

Here, we report the analysis of broad-scale patterns of species distribution and abundance based on recent meticulous search for plant species, including freshwater species, by numerous skilled botanists in voluntary atlas projects examining several hundred 5 km × 5 km quadrats covering large, representative proportions of Denmark and Southern Sweden. We used this general background to perform a statistical evaluation of relative occupancy of all freshwater species. We excluded a few recent immigrant species, which may not yet have filled their potential distribution and species of uncertain taxonomic status. We first characterize the five regions with respect to human population density, land use, climate as well as Alk and total phosphorus (TP) concentrations of a large representative number of lakes (>5 ha). We then determine the occupancy of freshwater species in the five regions according to plant height, trophic preference and life-form. Finally we evaluate the historical development of the freshwater flora in East-Denmark, West-Denmark, and Scania, which have all undergone habitat loss and cultural eutrophication as well as reduction of grazing and vegetation cutting on the banks of lakes and streams during the last century.

We tested three main hypotheses for the contemporary biota composition and historical change of the freshwater flora that derive from the results of recent studies of a large number of local lakes and streams (Tyler and Olsson, 1997; Riis and Sand-Jensen, 2001; Baattrup-Pedersen et al., 2008; Sundberg, 2014; Baastrup-Spohr et al., 2017; Sand-Jensen et al., 2017). We hypothesized that the occupancy of species of different preference for nutrients and light should change between regions and over time in correspondence to the altered environmental conditions as follows:

(1) Occupancy of small and oligotrophic species increases, while the occupancy of tall and eutrophic species decreases along the regional gradient of lower population density, farming intensity, and water TP concentrations.
(2) Occupancy of species with life-forms tolerant of turbid water increases, while occupancy of species with life-forms intolerant of turbid waters decreases along the regional gradient of higher water TP concentrations and turbidity.
(3) Occupancy of species has changed with the historical eutrophication in East-Denmark, West-Denmark, and Scania in a way resembling the pattern across the contemporary nutrient gradient between regions.

## Materials and Methods

### Geographical and Environmental Conditions

Geography, land use, and demographic data were taken from the national statistical bureaus of Denmark^1^ and Sweden^2^. All five regions are lowland regions (maximum elevation: 131–377 m a.s.l) with a similar climate (30-year annual mean air temperature: 5.9–8.1°C, and precipitation: 612–754 mm; **Table 1**). All five regions were rich in lakes (>5 ha) (0.015–0.13 lakes km^-2^), and although size of the largest lakes varied (344–17,300 ha, **Table 1**), this difference is not important to the presence of submerged plants which are mostly confined to the shallow parts of small and medium-sized lakes and streams. Land use was separated into rural, agricultural, forest and nature areas. The majority of forests are used for timber production and for that reason were not included in the nature area.

**Table 1:** Geographical data on the surveyed regions.

Region	Largest lake (ha)	Lake density (km^-2^)	Highest elevation (m)	Yearly precipitation (mm)	Yearly mean temperature (°C)	Summer mean temperature (°C)
West Denmark	1,713	0.015	173	754	7.6	15.1
East Denmark	4,072	0.020	131	612	8.1	15.8
Scania	5,400	0.024	212	748	7.2	15.4
Blekinge	344	0.130	189	676	6.9	15.2
Småland	17,300	0.083	377	747	5.9	14.6

*Lake density was calculated using the number of lakes (>5 ha) in each region based on GIS layers retrieved from (Danish data, http://www.miljoeportal.dk; Swedish data, http://www.smhi.se). Meteorological data (precipitation and temperature) were retrieved from the meteorological institutes of Denmark (http://www.dmi.dk) and Sweden (http://www.smhi.se).*

Danish data on TP and Alk in lake waters were collected as part of the national monitoring program (NOVANA, Lauridsen et al., 2005). We extracted data collected during the peak of the inventory period of the Atlas Flora Danica investigation (1995–2008) from lakes larger than 5 ha. Swedish data on TP and Alk in larger lakes (>5 ha) were collected from the national monitoring program^3^ in the period 2012–2016. Average site-specific measurements in surface waters were used in the statistical analyses.

### Species Occurrence Data

Species data were collected from recent regional floristic surveys in South Scandinavia (Fröberg, 2006; Edqvist and Karlsson, 2007; Tyler et al., 2007; Hartvig, 2015). In all four atlas projects, great care was taken to obtain an even coverage of examined quadrats across the region and to train and assist citizen scientists (i.e., very skilled amateur botanists) to ensure accurate species identification (e.g., Hartvig, 2015). We only included species that have occurred in the region for at least 100 years and, presumably, have attained a distribution, which corresponds with their biological traits. Thus, four species, which have recently appeared (*Azolla filiculoides*, *Callitriche obtusangula*, *Elodea nuttallii*, and *Lemna turionifera*) and still have very restricted distribution ranges were excluded together with two taxa of *Ranunculus penicillatus* (*ssp. pseudofluitans* and *ssp. penicillatus*), which are extremely rare, difficult to identify and have probably been over-looked in some regions. Differences in level of identification between surveys were handled by lumping subspecies and a limited number of problematic species (see Supplementary Information).

In all investigations, species incidence was scored as occupancy in grids of 5 km × 5 km cells, except in Scania where collection originally used a 2.5 km × 2.5 km grid size. Georeferenced primary data from Scania (obtained through GBIF) was resampled onto a 5 km × 5 km grid in a GIS environment [ArcGIS v. 10.5, with the national geodetic datum of Sweden (RT 1990) as specified by Tyler et al., 2007]. Owing to large differences in geology of the landscape of Denmark, this country was separated into a western part (Jutland) with a mixture of high-alkaline clayish soils and low-alkaline sandy soils, and an eastern part (East-Denmark), made up mainly by larger islands with predominantly alkaline, clayish soils. The smaller islands situated in the sea between Denmark and Sweden (Læsø, Anholt, and Bornholm) were left out, because of their unique geology and land use.

Several hundred grids were meticulously examined in each of the five regions. Within the grids, all standing waters (lakes and ponds: hereafter referred to as lakes) and running waters (rivers, streams, brooks, springs, canals and ditches: collectively streams) of different size were studied for the presence of aquatic plants. Although the exact number of freshwater localities is unknown it amounts to several thousand within each region. In Denmark, for example, there are about 100,000 lakes (the majority very small ponds) and about 64,000 km of streams (Sand-Jensen, 2013). Total number of lakes exceeding 0.01 ha is about 145 times larger than lakes exceeding 5 ha. Using this proportion, estimated total number of lakes within 5 km × 5 km grids is 55–470 between regions. Length of streams within 25 km^2^ grids is on the order of 35 km assuming the mean density in Denmark.

In each of the five regions, occurrence of individual species was converted to a proportion by dividing by the number of investigated grid cells. These frequencies of occurrences were used in the statistical analyses.

### Historical Changes

In three of the investigated regions (West-Denmark, East-Denmark, and Scania), earlier broad-scale investigations enabled assessment of species distributional changes during the course of a 50–80 years period. Species distributions obtained in the recent Danish investigation (Hartvig, 2015) were compared to a former similar project (Danish Topographical Botanical Investigation; Vestergaard and Hansen, 1989) carried out mainly in the period 1905–1927, and published as commented distribution maps over the subsequent decades. Due to differences in methodology, the distributional change over time was evaluated on an ordinal scale: *severe reduction* (>50% reduction in distribution or occurrence), *moderate reduction* (obvious but <50% reduction in distribution or occurrences), *little or no change*, *moderate increase* (50–100%), *strong increase* (>100% increase in distribution or occurrence) (Hartvig, 2015). Similarly in Scania, an investigation of the flora was conducted from 1938 to 1967 and an examination of the distributional changes of most species was published by Tyler and Olsson (1997), and analysis of all species was made available (Torbjörn Tyler pers. comm. 2016). The more fine-grained scale of change reported from Scania, was re-categorized to the same ordinal scale as used for the Danish data.

### Species Traits

To quantify the differential response by species or species groups, trait data regarding plant height, trophic prevalence and life form was compiled. Plant height was scored as maximal stem length for elodeid species (i.e., species with leaves scattered along an extended stem) and maximum leaf length for rosette species of the isoetid life form (i.e., species with leaves in rosette from a short stem; Schou et al. (2017). Trophic prevalence for TP of each species was scored as the ICM value by Kolada et al. (2014). Finally, each species was assigned to one of four life-form groups: (1) isoetids and creeping plants, (2) short elodeids (≤1 m tall), (3) tall elodeids (>1 m tall), and (4) floating-leaved plants (lemnids) using morphological descriptions in Schou et al. (2017). The submerged life-forms 1 to 3 represent a gradient of increasing height and are expected, when submerged at the same water depths, to exhibit increasing tolerance to turbid water by being able to grow toward the water surface. By extending the shoots, however, the ability to supply the plant with CO_2_ from the rich sediment source decreases because of longer diffusion path. Elodeid species are predominantly capable of efficient bicarbonate use to assist the diffusive entry of CO_2_ (Maberly and Spence, 1983). Floating-leaved plants of life-form 4 are independent of light attenuation and inorganic carbon supply in the water once their leaves have been placed at the water surface. Life-forms 1 to 4, therefore, represent increasing turbidity tolerance. When species in rare cases can develop different life-forms (e.g., *Juncus bulbosus* and *Potamogeton gramineus*) the prevalent life-form was applied. Life-form groups were included in the statistical analyses as a nominal variable to enable quantification of between group differences.

The assignment of species to height, trophic preference and life-form (and turbidity tolerance) is shown in Supplementary Table S1.

### Statistical Analysis

The potential difference in water chemistry variables (TP and Alk) was analyzed using Kruskal–Wallis test followed by Dunn’s *post hoc* test. To analyze how individual species traits were related to species frequency of occurrence in the investigated regions we used linear regression models with species frequency as the dependent variable and individual species traits as the explanatory variable (lm in R). Species frequency of occurrence was logit-transformed prior to analysis. As the relationship between traits and frequency of occurrence could change between regions, they were included as a covariate. Significant interactions between regions and traits, as tested by likelihood ratio tests, were interpreted as changing trait-frequency relationships. In case of a significant interaction, we made pairwise contrasts between regions to test which regions had significantly different trait-frequency relationships using the lstrends function in the lsmeans-package (R Development Core Team, 2015).

The historical changes of species occurrence were measured on an ordinal scale (severe reduction, moderate reduction, etc.) and analyzed using ordinal logistic regression (Venables and Ribley, 2002). Similar to the analyses of the current frequency of occurrence (see above), we tested for significant interactions between region and frequency to evaluate whether trait-frequency relationships changed between regions. Because significant interactions were not observed no contrast models were applied. Ordinal regression was performed using the clm2 function using the ordinal-package (R Development Core Team, 2015).

## Results

### Geographical and Environmental Conditions

**FIGURE 1:**
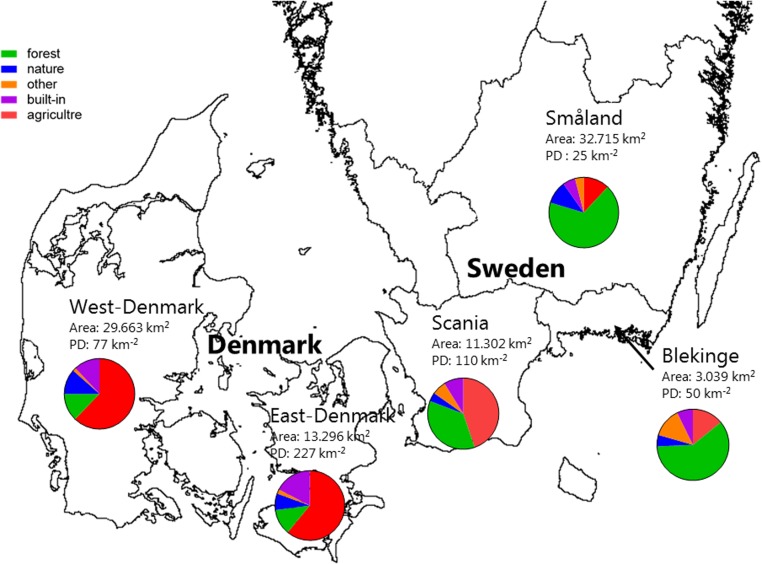
A map of the five Danish and South-Swedish regions denoting surface area and population density. The pie charts show the proportions of different land use categories within each region. The category “forest” contains all wooded areas (in the region primarily plantation forest), “nature” is open habitats along with lakes and rivers, “built in” is roads, buildings and other artificial surfaces, “agriculture” is farmed land and areas with livestock while “other” refers to areas not covered by any of the other categories (e.g., mines and golf courses).

**FIGURE 2:**
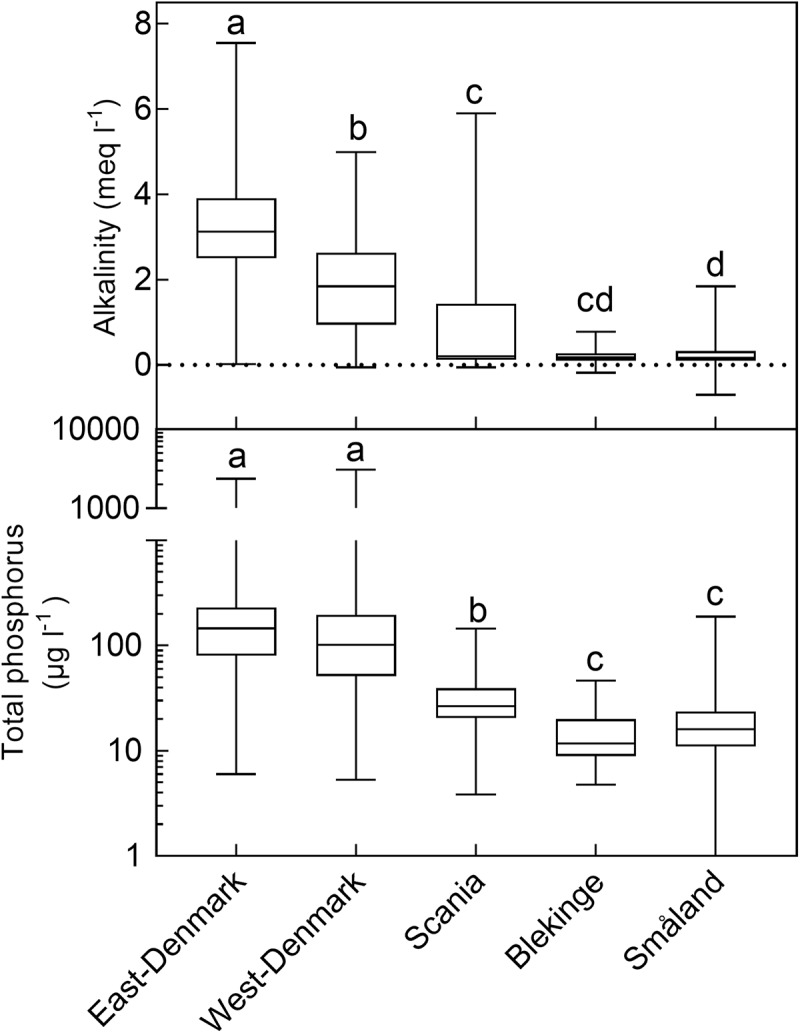
Box–Whiskers plots showing the distribution of alkalinity (upper) and total phosphorus (lower) in lake waters of the five Danish and South-Swedish regions. Boxes include 25–75 percentiles and the median, while whiskers extend to the data range. Lower-case letters indicate significant (*p* < 0.05) differences according to a Kruskal–Wallis test followed by a Dunn’s *post hoc* test.

There was a ten-fold difference in population density and a five-fold difference in landscape proportion of arable land along the geographical gradient from East-Denmark via West-Denmark, Scania and Blekinge to Småland, while forest cover showed a six-fold difference in the opposite direction (**Figure 1**). Forests in Denmark and Southern Sweden are subject to intense forestry, but not to fertilization. These gradients of population density and farming intensity from Denmark to Småland, combined with much lower natural fertility of soils toward the East, cause lower nutrient input from domestic sewage, agricultural fields and nature areas and may account for the significant (Kruskal–Wallis, *p* < 0.0001) 10-fold difference in in-lake median TP concentrations across the study area (**Figure 2**, upper). The majority of Danish lakes are eutrophic and hypertrophic (25–75 percentiles: 80–230 μg TP L^-1^) and few approach the oligotrophic threshold (10 μg TP L^-1^). Toward West-Denmark the concentration tends to decrease (Dunn’s test, *p* = 0.058) and when moving to the Southern Swedish region of Scania the concentration decreases significantly (Dunn’s test, *p* < 0.0001, **Figure 2**, upper). The majority of Småland lakes are nutrient poor (25–75 percentiles: 11.0–23.6 μg TP L^-1^) and some reach the supra-oligotrophic level (1 μg TP L^-1^). Blekinge resembles Småland in water phosphorus concentration (Dunn’s test, *p* = 0.18) and also with respect to population density and land use.

Most lakes in East-Denmark, West-Denmark, and Scania are hardwater lakes in accordance with the dominance of calcareous soils, however, with significantly decreasing Alk from East-Denmark over West-Denmark to Scania (Kruskal–Wallis, *p* < 0.0001, Dunn’s test, *p* < 0.001). Most lakes in Blekinge and Småland are softwater lakes in accordance with the dominance of sandy soils and silicious bedrocks. Alk is significantly lower compared to East-Denmark and West-Denmark (Dunn’s test, *p* < 0.001), while Småland but not Blekinge is significantly different from Scania (Dunn test, *p* = 0.073, **Figure 2**, lower). The range of alkalinities is extensive in all five regions, however, with some softwater lakes being present in East-Denmark, West-Denmark, and Scania and some hardwater lakes in Blekinge and Småland. Medium Alk and TP are highly correlated across regions (Pearson *R* = 0.99, *p* < 0.001).

### Species Pool and Similarity of Species Occupancy

The five regions resembled each other closely in terms of species composition (Sørensen similarity: 81–94%, **Table 2**), while Bray–Curtis similarity accounting for species abundance were markedly different between regions. East-Denmark, West-Denmark, and Scania (73–83%) formed one group and Blekinge and Småland (82%) another group of high internal similarity. Bray–Curtis similarity was low (46–57%) in the comparison of East-Denmark and West-Denmark with Blekinge and Småland (**Table 2**). Scania had a medium Bray–Curtis similarity (66–68%) to neighboring Blekinge and Småland.

**Table 2:** Presence-absence based similarity (Sørensen-similarity) and occupancy based similarity (Bray–Curtis similarity, in bold) in aquatic macrophyte community composition among regions.

	East-Denmark	West-Denmark	Scania	Blekinge	Småland
East-Denmark	–	**75.7**	**72.6**	**49.3**	**45.8**
West-Denmark	84.6	–	**82.8**	**57.3**	**56.7**
Scania	84.9	93.3	–	**67.6**	**65.9**
Blekinge	81.2	82.7	87.1	–	**81.6**
Småland	81.1	90.9	93.8	88.6	–

### Occurrence and Plant Height

**FIGURE 3:**
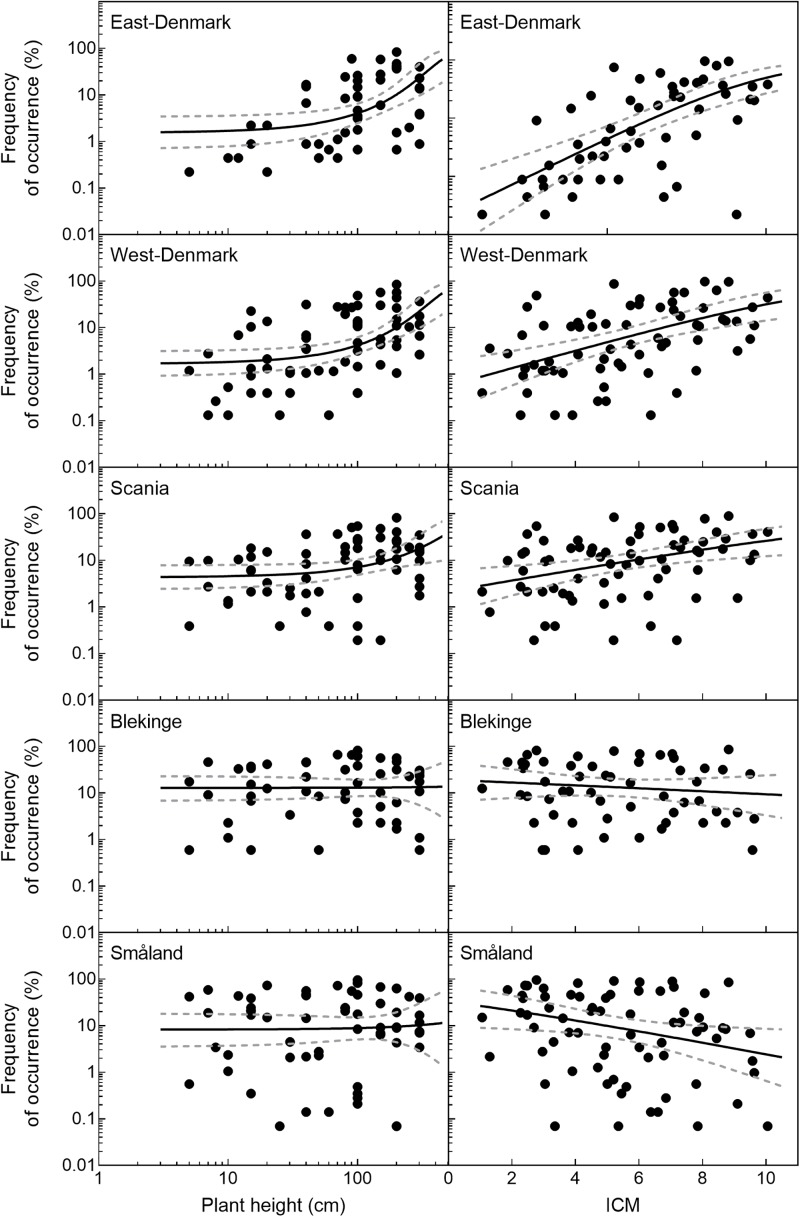
Frequency of occurrence of individual species as a function of their height and trophic preference (ICM-values) in the five Danish and South-Swedish regions. Regression lines and their confidence intervals are linear models based on logit transformed species frequencies. There was a significant positive relationship between plant height and frequency (*p* < 0.05) in East-, West-Denmark, and Scania, but not in Blekinge and Småland. For ICM there was a significant positive relationships between plant height and frequency (*p* < 0.05) in East-, West-Denmark, and Scania and a significant negative relationship in Småland (*p* < 0.05). In Blekinge the relationship between ICM and frequency was not significant (*p* > 0.05).

The relationship of species occurrence to plant height changed significantly across the five regions (likelihood ratio test of interaction between region and plant height, *p* = 0.012, **Figure 3**). There was a significant positive relationship between plant height and frequency of occurrence in the alkaline and nutrient-rich regions of East-Denmark, West-Denmark, and Scania, while the relationship was not significant in the nutrient-poor and low-alkaline regions of Blekinge and Småland (**Table 3**). Pairwise contrasts of the slopes of the regression line of species occurrence *versus* plant height tended to be steeper in East-Denmark and West-Denmark (*p* < 0.1) compared with lower values close to zero in Blekinge and Småland (**Table 3**). Thus, the mean plant height of submerged species weighted according to their occurrence decreased gradually from 98 cm in East-Denmark to 77 cm in Småland (**Table 3**).

**Table 3:** Occupancy weighted mean height in the different regions along with the relationship between occupancy and plant height of individual species in the five regions as tested by linear models on logit transformed occupancy data.

			Pairwise contrasts (*p*-value)			
	Weighted mean plant height (cm)	*R*^2^ (*p*-value)	East-Denmark	West-Denmark	Scania	Blekinge
East-Denmark	97.8	0.22 (>0.001)				
West-Denmark	89.8	0.21 (>0.001)	0.99			
Scania	90.3	0.09 (0.016)	0.71	0.75		
Blekinge	86.6	0.0 (0.95)	0.074	0.068	0.60	
Småland	76.8	0.0 (0.80)	0.099	0.092	0.70	0.99

*Also, pairwise contrasts in the relationship between occupancy and plant height among regions are specified by p-values. Significant changes (p < 0.05) indicates that the relationship between occupancy and plant height are changing between regions.*

### Occurrence and Trophic Prevalence

Occurrence of all freshwater species, including submerged and floating-leaved forms of different trophic prevalence for phosphorus, changed significantly across the five regions (**Figure 3**) in accordance with falling phosphorus concentrations in the waters (**Figure 2**). There was a significant positive relationship between plant species’ trophic preference (ICM) and occurrence in East-Denmark, West-Denmark, and Scania (linear regression, *p* < 0.05), while Blekinge showed no relationships and Småland showed a significant inverse relationship with species of low phosphorus preference being more abundant than species of high phosphorus preference (**Table 4**). Thus, the mean trophic preference of all species weighted according to their occurrence differed gradually from 6.4 in East-Denmark to only 4.3 in Småland (**Table 4**). Among submerged freshwater plants there was a significant positive relationship between plant height and phosphorus preference, though the correlation was weak (Pearson-R = 0.4, *p* < 0.05).

**Table 4:** Occupancy weighted mean ICM-values (Kolada et al., 2014) in the different regions along with the relationship between occupancy and ICM-value of individual species in the five regions as tested by linear models on logit transformed occupancy data.

			Pairwise contrasts (*p*-value)			
	Weighted mean ICM	*R*^2^ (*p*-value)	East-Denmark	West-Denmark	Scania	Blekinge
East-Denmark	6.4	0.37 (>0.001)				
West-Denmark	5.6	0.22 (>0.001)	0.8233			
Scania	5.5	0.12 (0.003)	0.2397	0.8108		
Blekinge	4.5	0.01 (0.44)	0.0005	0.0074	0.1400	
Småland	4.3	0.07 (0.027)	<0.0001	<0.0001	0.0012	0.6427

*Also, pairwise contrasts in the relationship between occupancy and ICM-values among regions are specified by p-values. Significant changes (p < 0.05) indicates that the relationship between occupancy and ICM-value are changing between regions.*

### Occurrence and Life-Form

Life-forms of freshwater species were divided into four categories (1–4) which are expected to represent a gradient of increasing tolerance to light attenuation in the water (i.e., turbidity tolerance) and increasing preference for nutrient richness. The three submerged life-form categories have increasing plant height and trophic preference to phosphorus, and among floating-leaved forms unrooted species rely solely on nutrients from the water. Thus, mean ICM values shifted among life-forms in the order: 4.0 (life-form 1, isoetids), 4.9 (life-form 2, short elodeids), 6.4 (life-form 3, tall elodeids) and 7.4 (life-form 4, floating-leaved species).

**FIGURE 4:**
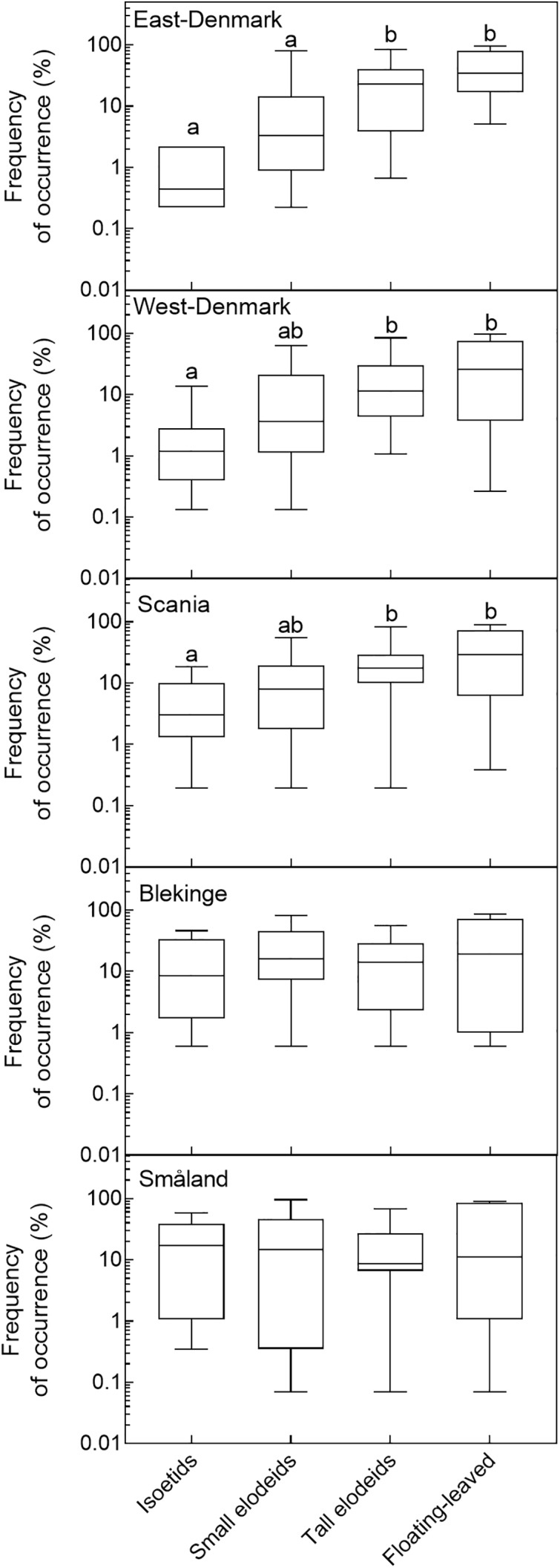
Box–whisker plots based on frequency of occurrence of individual species within the analyzed life-form groups in the five Danish and South-Swedish regions. Boxes include 25–75 percentiles and the median, while whiskers extend to the data range. Lower-case letters indicate significant (α = 0.05) differences according to a Kruskal–Wallis test followed by a Dunn’s *post hoc* test.

**FIGURE 5:**
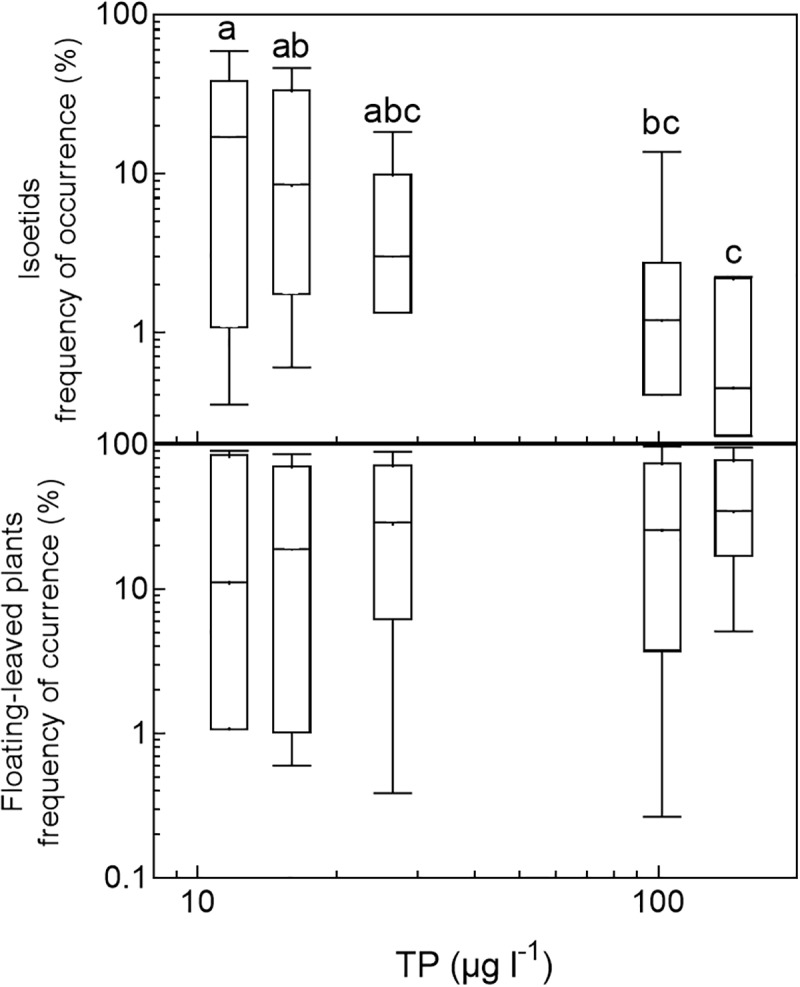
Box–whisker plots based on frequency of occurrence of individual species within selected life-form groups (isoetids and floating-leaved plants) plotted against the median phosphorus concentration in the five analyzed regions. Boxes include 25–75 percentiles and the median, while whiskers extend to the data range. Lower-case letters indicate significant (α = 0.05) differences according to a Kruskal–Wallis test followed by a Dunn’s *post hoc* test.

In the three nutrient-rich and alkaline regions (East-Denmark, West-Denmark, and Scania), median occupancy differed markedly from life-form 1 to 4 (Kruskal–Wallis, *p* < 0.01), whereas a more even occupancy of life forms was observed in the two nutrient-poor, low-alkaline regions (Blekinge and Småland, Kruskal–Wallis, *p* > 0.4, **Figure 4**). Differences between regions in occupancy were particularly strong for isoetids, which had a 50-fold higher median occupancy in Småland (20%) than in East-Denmark (0.4%, Kruskal–Wallis, *p* = 0.002, Dunn’s test, *p* = 0.02) along the TP gradient (**Figure 5**). Floating-leaved species showed the opposite pattern, decreasing in median occupancy from 30 to 10% along the gradient, though the trend was not significant (Kruskal–Wallis, *p* = 0.08).

### Historical Flora Changes

**FIGURE 6:**
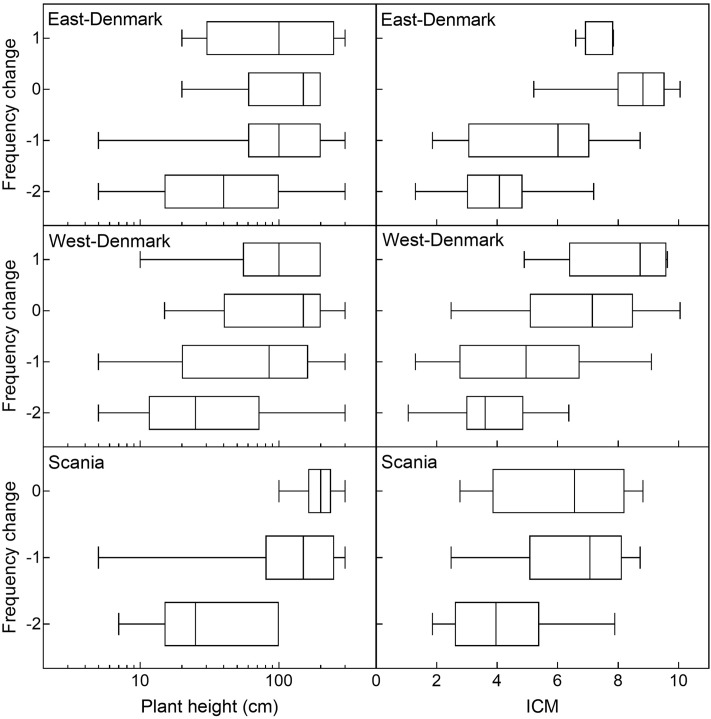
Historical ordinal change of individual species as a function of plant height and species trophic preference (ICM-values) in East-, West-Denmark, and Scania. Due to a very low sample size (*n* < 4) the class of marked increase (+2) of the Danish regions and both increase groups in Scania (+1, +2) were left out of the graph. There was a positive significant relationship between both plant height and ICM vs. historical change (ordinal logistic regression, *p* < 0.001) indicating that declining species are small and with preference for oligotrophic habitats, while stabile or increasing species are larger with more eutrophic preferences. There was no significant interaction between plant height and region (likelihood ratio test *p* = 0.12) or ICM and region (likelihood ratio test *p* = 0.75) indicating that the relationships did not differ between regions.

Historical changes in species abundance during the 20th century in East-Denmark, West-Denmark, and Scania were significantly correlated (Spearman-R: 0.59–0.73, *p* < 0.0001) and the average change for all three regions was significantly correlated to the pattern observed along the spatial gradient of increasing TP concentrations from Småland to Denmark (Spearman-R: 0.57, *p* < 0.001). There was a significant positive relationship (ordinal logistic regression, *p* < 0.001) between plant height and classes of historical changes showing that smaller species declined while larger species changed less and in few cases increased (**Figure 6**). The interaction between region and plant height was not significant (likelihood ratio test, *p* = 0.12), indicating that the relationship between historical changes and plant height did not differ between regions.

The relationship between trophic preference (ICM) and historical change was very similar with no significant interaction (likelihood ratio test, *p* = 0.75) but showed a highly significant positive effect of ICM on historical change (ordinal logistic regression, *p* < 0.001, **Figure 6**).

To analyze the relationship between life-form groups and species’ historical changes, we tested if the median historical change differed between life-forms in each region. In all three regions, the median historical change differed significantly between life-form groups (Kruskal–Wallis, *p* < 0.01). Also, in all regions, small isoetids declined more than tall elodeids and floating-leaved plants (Dunn’s test, *p* < 0.05). Furthermore, in Scania small elodeids also declined significantly more compared to tall elodeids and floating-leaved plants (Dunn’s test, *p* < 0.05).

## Discussion

The geographical occupancy of aquatic plant species changes across South Scandinavia in response to vast differences in nutrient levels, which express a spatial version of a temporal gradient observed during anthropogenic eutrophication in three of the regions. These parallel spatial and temporal directional changes in species occupancy are significantly related to the species’ life-form and adaptations to nutrient and light availability, implying that trait–environment interactions, rather than biogeography, are responsible for the patterns of species occupancy. The observed predominant role of species traits for occupancy at large regional scales accords with the patterns observed across spatial and historical nutrient gradients in individual lakes. We discuss the importance of these nutrient-driven patterns on species occupancy. We include the role of Alk, which is closely correlated to TP across the regional gradient, and the role of historically reduced disturbance of lake banks by ceased livestock grazing, mowing and reed cutting as well as controlled water level fluctuations. Finally, we discuss how re-oligotrophication and re-establishment of cattle grazing and water level fluctuations might halt and reverse the historical decline of biodiversity, in general, and the loss of red-listed, oligotrophic isoetids and small elodeids, in particular.

### Spatial and Temporal Species Development

A key result of the present study is the profound changes in species occupancy between the five South-Scandinavian regions. We found a predominance of tall submerged elodeids and floating-leaved species of high nutrient prevalence in the most densely populated and intensively farmed regions in Denmark. This composition contrasted the more even occupancy of life-forms and the significantly lower mean nutrient prevalence of species assemblages in Swedish Blekinge and Småland, with low human population density and low farming intensity. The most distinctive contrast was the 50-fold higher occupancy of small, oligotrophic isoetid species in Småland compared with East-Denmark accompanying a 10-fold lower median TP concentration.

Another key result was the overall agreement between the regional difference of species occupancy *versus* nutrient richness and the temporal development of species occupancy in West-Denmark and East-Denmark and in Scania during the last century. Over this time period, numerous freshwater bodies have undergone marked eutrophication (Sand-Jensen et al., 2000; Jensen et al., 2017) as a result of several-fold increase in nitrogen and phosphorus surplus on farmland (Kyllingsbæk, 2008) and, up to 1980–1990, higher outlet of nutrient-rich domestic sewage (Kronvang et al., 2006) and greater nitrogen deposition from the atmosphere (Galloway et al., 2004; European Environment Agency, 2014). The historical development was a marked decline of submerged species of low plant height and low phosphorus preference (i.e., mainly isoetids and small elodeids), and a much smaller change of tall submerged and nutrient tolerant species (i.e., mainly tall elodeids and floating-leaved plants). Examples of isoetids, which have decreased markedly in abundance, are *Lobelia dortmanna*, *Isoëtes echinospora*, *I. lacustris*, *Pilularia globulifera*, and *Subularia aquatica.* All five species have vanished from East-Denmark (Schou et al., 2017). Isoetids as a group, and the five mentioned species in particular, have also decreased during the last century in Central Sweden (Rydberg and Wanntorp, 2001; Maad et al., 2009), the Netherlands and Northern Germany (Arts, 2002), emphasizing the generality and wide-scale character of the change. Very few freshwater plant species have obtained higher occupancy during the last century in Denmark and Scania, but among them are elodeids of high trophic preference (e.g., *Elodea canadensis*, *Ceratophyllum demersum*, and *C. submersum*). Among the most common freshwater species in all regions are three floating-leaved species, which have maintained high occupancies throughout the last century (*Lemna minor*, *Persicaria amphibia*, and *Potamogeton natans*).

The composition and the historical changes to the local floras drive the spatial and historical patterns of species occupancy observed at regional scales. From pristine conditions in 1911, Lake Fure in East-Denmark experienced 70 years of steeply increasing TP concentration (25 to 600 μg P L^-1^) and decreasing Secchi transparency (5.5–1.7 m). While the original single isoetid and 11 small elodeid species disappeared, all nine tall elodeid and four rooted floating-leaved species survived (Sand-Jensen et al., 2017). In parallel to this, the mean trophic prevalence of the macrophyte community increased by 2.1 ICM-units (5.9–8.0), similar to the regional change from Småland to East-Denmark (4.3–6.4).

### Other Environmental Factors Influencing Species Occupancy

The fact that changes in species occupancy, ICM-values, plant height and life-forms followed the same course regionally and temporally strongly supports the conclusion that changes in trophy were the main determinants of flora changes. Whereas TP and Alk were closely correlated across regions, historical changes of TP and vegetation always take place without changes to Alk. The issue of Alk is relevant because many elodeids use bicarbonate (a close proxy of Alk) in photosynthesis, whereas it is unavailable to isoetids and floating-leaved plants, which – in addition to CO_2_ in the water – predominantly use CO_2_ from the rhizosphere and atmosphere, respectively (Madsen and Sand-Jensen, 1991). Although some elodeid species (e.g., *Stuckenia pectinata*) are apparently dependent on a certain level of bicarbonate to grow well (e.g., Sand-Jensen, 1983; Madsen and Sand-Jensen, 1987), all elodeid species are found across a broad bicarbonate range and certain species (e.g., *Myriophyllum alterniflorum* and *Potamogeton alpinus*) grow at bicarbonate concentrations close to zero (Vestergaard and Sand-Jensen, 2000). Thus, there are differences in occupancy of individual elodeid species between low and high alkaline regions, though not any major regional differences in the occupancy of the elodeid life-forms.

Historical eutrophication during the last century cannot be treated as a single mechanism, however, because it was accompanied by complex changes in disturbance regimes. Traditional agricultural practice included cattle grazing and hay making on lake shores and along streams (Fritzbøger, 2004; Vellend et al., 2017). These practices may well have mimicked natural disturbances. In addition, natural water level fluctuations have also been altered in many lakes and along streams, including both water level stabilization, artificially large daily to yearly fluctuations and unnatural timing of fluctuations (Wantzen et al., 2008; Mjelde et al., 2013; Bejarano et al., 2017). In sum, the ceased disturbances probably contributed to control the dominance of tall amphibious and emergent species in the historical landscape, and conversely contributed to benefit competitively inferior species, such as isoetids and small elodeids on lake and stream banks.

Eutrophication enhances density and height of tall emergent plants (Björk, 1967), thereby increasing their ability to competitively exclude slow-growing isoetids and small elodeids (Keddy et al., 1994; Sand-Jensen, 1997). Together the described changes in agricultural practice and eutrophication have been responsible for a 70% decrease in the presence of oligotrophic open lake shores formerly dominated by short vegetation in Uppland (Maad et al., 2009). Higher nutrient concentrations in lake waters also stimulate the growth of tall elodeid and floating-leaved species of high nutrient preference, because of their ability to place their leaves close to or on the lake surface and exert a strong shading effect on the bottom-dwelling vegetation (Baastrup-Spohr et al., 2017; Murphy et al., 2017; Sand-Jensen et al., 2017).

### Predominant Role of Species Traits

While the total species pool was qualitatively very similar in the five South-Scandinavian regions, species occupancy patterns varied markedly in accordance with species traits and regional differences in eutrophication and land use. Over the past one or two centuries, there has been a directional change in species abundance patterns in the most intensely cultivated regions of East-Denmark, West-Denmark, and Scania, deflecting the floras away from the historical abundance patterns that resembled those still found in the least influenced regions, Blekinge and Småland. Isoetid species were much more common in Denmark and Scania in the period 1880–1930, also in high-alkaline lakes (Odgaard, 1998; Sand-Jensen, 2001; Pedersen et al., 2006), emphasizing that historical changes in land use, agricultural practice and eutrophication have increased the regional divergence of occupancy of species and life-form groups. Thus, environmental changes and differences in species traits can account for the contemporary patterns of species occupancy, whereas differences in purely biogeographical processes cannot to nearly the same degree. However, the influence of biogeography should be small in the first place, considering the restricted geographical di*s*tance (i.e., less than 400 km from central West-Denmark to central Småland) and the similar Holocene vegetation history (Cowling et al., 2001; Giesecke et al., 2017; Marquer et al., 2017). The comparisons with Central Sweden (Maad et al., 2009) and lakes in North-West Europe (Roelofs, 1983; Arts, 2002) suggest that the conclusion can be extended to a greater part of Europe having a similar species pool and historic development of the freshwater environment and flora.

Plant height and trophic preference are efficient predictors of the historical changes to species preference in the freshwater flora in South Scandinavia, much the same way as has been demonstrated for the terrestrial flora in Denmark, Scania, and Central Sweden (e.g., Tyler and Olsson, 1997; Maad et al., 2009; Sundberg, 2014; Hartvig, 2015). We used ICM-values to characterize the trophic preference (mainly phosphorus) of freshwater species because they are based on extensive data (Kolada et al., 2014) and are provided at a continuous scale rather than the ordinal scale of Ellenberg-N values. However, the two indices are closely correlated (Kolada et al., 2014) and application of Ellenberg-N values for the freshwater flora lead to qualitatively similar conclusions as ICM-values (data not shown). The decline of isoetids and short elodeids also has a parallel in the historical decrease of short light-demanding terrestrial species characteristic of the open vegetation in the nutrient-poorer historical terrestrial landscape (Walker and Preston, 2006; Keith et al., 2009; Saar et al., 2012; Tyler et al., 2018).

Despite the usefulness of life-form, plant height and trophic preference, there is considerable variability in the occupancy and historical development of species of apparent similarity. The variability unaccounted for may be due to species traits that were not considered. While plant height as a maximum standard value can account for competitive ability in a dense communities and ICM-values for phosphorus prevalence, the real competitive ability of a species is determined by unknowns, i.e., its realized vertical and lateral growth in a given nutrient and light environment. Likewise, the rates of growth and dispersal as well as the tolerances to extreme weather and physical disturbance are unaccounted for, though they probably show important interspecific differences. For example, *Plantago uniflora* has a higher growth rate and lateral vegetative dispersal by runners than most other isoetid species (Sand-Jensen and Søndergaard, 1978) and can maintain a higher occupancy in phosphorus-rich regions. A more fine-grained analysis is thus possible once experimentally established quantitative measures of the mentioned species traits become available.

### Environmental Improvements and Restoration

The deterministic relationships between environmental conditions and occupancy of species in accordance with their traits may direct action plans with the aim to restore the freshwater flora and protect rare and threatened species. Early programs in Denmark and Sweden and the later EU Water Frame Directive and Habitat Directive over the last two to three decades have had the goal of restoring the ecological quality of nature habitats by reducing the excessive historical eutrophication. Re-colonization of species and recovery of community composition are greatly delayed when improvements of physico-chemical conditions (e.g., water and sediment chemistry) are slow, and dispersal capacity is low because species only survive in low numbers in distant refugia. This is the situation for isoetids in East-Denmark, in which nutrient levels are high and isoetids are rare or have gone extinct (Sand-Jensen et al., 2017). It is difficult to improve the quality of relatively nutrient-poor lakes in regions with nutrient-rich soils, high farming intensity and substantial atmospheric nutrient deposition (Baastrup-Spohr et al., 2017). It is easier to improve nutrient conditions in eutrophic lakes and promote recolonization of nutrient-tolerant elodeids (Baastrup-Spohr et al., 2017; Murphy et al., 2017).

Establishment of a rich flora, including isoetids and short elodeids, is even possible under nutrient-rich conditions, provided that the lake shore is kept open by grazing and physical exposure removing tall amphibious and emergent species and allowing colonization of small species from neighboring sites. This is the scenario in the restored Lake Filsø established on fertile farmland in West-Denmark (Baastrup-Spohr et al., 2016a). Strong winds and shallow waters have rapidly reduced the organic content of the lake shores making them suitable to aquatic plant colonization assisted by dense populations of water birds as dispersal vectors (Kragh et al., 2017).

Water level fluctuations of intermediate size (1–3 m annually) can support high aquatic plant biodiversity (Rørslett, 1991). Growth of isoetids can be markedly stimulated by exposure to air, as shown for *Plantago uniflora* (Robe and Griffiths, 1998; Baastrup-Spohr et al., 2016b). Locally rare isoetid species, such as *Subularia aquatica* and *Ranunculus reptans*, are tolerant of harsh winter drawdowns (Mjelde et al., 2013). Enforcement of natural water level fluctuation is, therefore, probably a means of securing viability of populations of these threatened species. In fertile habitats, disturbance by grazing or physical disturbances may be necessary to control tall competitors on the lake banks, and thus to ensure long-term survival of the low-growing species.

In summary, we could predict parallel regional and historical changes in freshwater floras across nutrient gradients, through the use of species traits (e.g., plant height, trophic prevalence and life-form strategies), which have previously been applied successfully to changes in terrestrial floras. We identified a need to include additional species traits in future fine-grained analyses in order to better account for dispersal potential, competitive ability and tolerance of physical perturbation. Recovery obtained through re-oligotrophication, grazing of banks and physical perturbation may follow a trajectory toward greater abundance of threatened species and overall higher richness, albeit probably with a prolonged time delay in relatively eutrophic regions.

## Author Contributions

KS-J, LB-S, and HB developed the idea. PH, JS, and DC organized the Danish data and KS-J, PH, and LB-S extracted the remaining data. LB-S, KS-J, TN, and DC analyzed the data. LB-S performed the statistical analysis. KS-J wrote the paper with assistance from HB and LB-S. All authors commented on an earlier draft of manuscript.

## Conflict of Interest Statement

JS was employed by company BioPix.dk. The other authors declare that the research was conducted in the absence of any commercial or financial relationships that could be construed as a potential conflict of interest.

## Footnotes

1 www.dst.dk

2 www.scb.se

3 http://miljodata.slu.se/mvm/
