# Supplementary material for: The Dangers of Being a Small, Oligotrophic and Light Demanding Freshwater Plant across a Spatial and Historical Eutrophication Gradient in Southern Scandinavia
Source: Front Plant Sci. 2018 Feb 2;9:66. doi: 10.3389/fpls.2018.00066 (PMC5801560; doi:10.3389/fpls.2018.00066)
Supplement: Supplementary file 1 [file Table_1.docx]

Supplementary Material

**The dangers of being a small, oligotrophic and light demanding freshwater plant across a spatial and historical eutrophication gradient in Southern Scandinavia**

Kaj Sand-Jensen, Hans Henrik Bruun, Tora Finderup Nielsen, Ditte Marie Christiansen, Per Hartvig, Jens Christian Schou and Lars Baastrup-Spohr

***Correspondence:**

Professor Kaj Sand-Jensen

ksandjensen@bio.ku.dk

# Supplementary Data

Table S1. The species included in the study along with their life-form (see materials and methods), maximal plant height according to Schou et al. (2017) and ICM values extracted from Kolada et al. (2014). We used only the normal maximal plant heights and not the extreme heights (noted in parenthesis for some species) in Schou et al. (2017).

| Taxon | Life-form | Plant height | ICM-value |
| --- | --- | --- | --- |
| *Baldellia ranunculoides* | 1 | 20 | 4.9 |
| *Baldellia repens* | 1 | 15 |  |
| *Butomus umbellatus* | 3 | 100 | 8.73 |
| *Callitriche brutia* | 2 | 30 |  |
| *Callitriche cophocarpa* | 2 | 40 | 5.1 |
| *Callitriche hamulata* | 2 | 80 |  |
| *Callitriche hermaphroditica* | 2 | 30 | 6.29 |
| *Callitriche palustris* | 2 | 40 | 3.6 |
| *Callitriche platycarpa* | 3 | 200 | 10.05 |
| *Callitriche stagnalis* | 2 | 40 | 5.99 |
| *Catabrosa aquatica* | 4 |  |  |
| *Ceratophyllum demersum* | 3 | 300 | 7.82 |
| *Ceratophyllum submersum* | 3 | 200 | 7.85 |
| *Crassula aquatica* | 1 | 5 | 3.04 |
| *Elatine hexandra* | 1 | 8 | 4.96 |
| *Elatine hydropiper* | 1 | 10 | 4.9 |
| *Elatine triandra* | 1 |  | 5.2 |
| *Eleocharis acicularis* | 1 | 15 | 4.78 |
| *Elodea canadensis* | 3 | 200 | 7.42 |
| *Groenlandia densa* | 2 | 65 |  |
| *Helosciadium inundatum* | 2 | 100 | 5.6 |
| *Hippuris vulgaris* | 2 | 80 | 4.49 |
| *Hottonia palustris* | 2 | 90 | 6.67 |
| *Hydrocharis morsus-ranae* | 4 |  | 7.09 |
| *Isoëtes echinospora* | 1 | 15 | 2.48 |
| *Isoëtes lacustris* | 1 | 15 | 2.35 |
| *Isolepis fluitans* | 2 | 40 | 1.3 |
| *Juncus bulbosus* | 2 | 100 | 2.77 |
| *Lemna gibba* | 4 |  | 9.63 |
| *Lemna minor* | 4 |  | 8.82 |
| *Lemna trisulca* | 2 |  | 8.44 |
| *Limosella aquatica* | 1 | 10 | 3.9 |
| *Lobelia dortmanna* | 1 | 7 | 1.86 |
| *Luronium natans* | 4 |  |  |
| *Lythrum portula* | 1 | 15 | 4.53 |
| *Montia fontana* | 2 | 15 |  |
| *Myriophyllum alterniflorum* | 3 | 200 | 3 |
| *Myriophyllum sibiricum* | 3 |  | 5 |
| *Myriophyllum spicatum* | 3 | 300 | 7.3 |
| *Myriophyllum verticillatum* | 3 | 150 | 5.74 |
| *Najas flexilis* | 2 | 25 | 3.35 |
| *Najas marina* | 2 | 50 | 6.78 |
| *Nuphar lutea* | 4 |  | 7.05 |
| *Nuphar pumila* | 4 |  | 4.7 |
| *Nymphaea alba* | 4 |  | 6.02 |
| *Nymphoides peltata* | 4 |  | 7.8 |
| *Oenanthe fluviatilis* | 3 | 200 |  |
| *Persicaria amphibia* | 4 |  | 8.07 |
| *Pilularia globulifera* | 1 | 30 | 3.31 |
| *Plantago uniflora* | 1 | 12 |  |
| *Potamogeton acutifolius* | 2 | 100 |  |
| *Potamogeton alpinus* | 3 | 250 | 4.13 |
| *Potamogeton berchtoldii* | 2 | 100 | 5.73 |
| *Potamogeton coloratus* | 2 | 70 |  |
| *Potamogeton compressus* | 2 | 100 | 5.45 |
| *Potamogeton crispus* | 3 | 200 | 8.02 |
| *Potamogeton friesii* | 2 |  | 5.35 |
| *Potamogeton gramineus* | 2 | 80 | 3.17 |
| *Potamogeton lucens* | 3 | 300 | 6.01 |
| *Potamogeton natans* | 4 |  | 5.21 |
| *Potamogeton obtusifolius* | 2 |  | 7.89 |
| *Potamogeton perfoliatus* | 3 | 300 | 4.95 |
| *Potamogeton polygonifolius* | 2 | 70 | 2.48 |
| *Potamogeton praelongus* | 3 | 300 | 4.08 |
| *Potamogeton pusillus* | 2 | 100 | 9.1 |
| *Potamogeton rutilus* | 2 | 60 | 6.37 |
| *Potamogeton trichoides* | 2 | 100 | 7.19 |
| *Ranunculus aquatilis* | 3 | 150 |  |
| *Ranunculus baudotii* | 3 | 300 |  |
| *Ranunculus circinatus* | 2 | 100 | 6.85 |
| *Ranunculus hederaceus* | 1 | 20 | 9.07 |
| *Ranunculus peltatus* | 3 | 300 |  |
| *Ranunculus reptans* | 1 | 5 | 3.04 |
| *Sagittaria latifolia* | 2 | 60 |  |
| *Sagittaria sagittifolia* | 3 | 200 | 6.71 |
| *Sparganium angustifolium* | 3 | 150 | 2.69 |
| *Sparganium emersum* | 3 | 150 | 7.1 |
| *Sparganium erectum* | 3 | 200 |  |
| *Sparganium glomeratum* | 4 |  |  |
| *Sparganium gramineum* | 3 | 300 | 3.8 |
| *Sparganium natans* | 2 | 100 | 4.07 |
| *Spirodela polyrhiza* | 4 |  | 9.57 |
| *Stratiotes aloides* | 2 | 40 | 6.6 |
| *Stuckenia filiformis* | 2 | 50 | 2.96 |
| *Stuckenia pectinata* | 3 | 200 | 8.64 |
| *Subularia aquatica* | 1 | 7 | 2.27 |
| *Utricularia australis/vulgaris* | 2 | 100 | 3.86 |
| *Utricularia intermedia* | 2 | 20 | 2.41 |
| *Utricularia minor* | 2 | 40 | 2.32 |
| *Utricularia ochroleuca/stygia* | 2 | 20 | 1.06 |
| *Zannichellia palustris* | 3 | 150 | 9.5 |

**References**

Kolada, A., Willby, N., Dudley, B., Nõges, P., Søndergaard, M., Hellsten, S., et al. (2014). The applicability of macrophyte compositional metrics for assessing eutrophication in European lakes. *Ecological Indicators* 45**,** 407-415. doi: <http://dx.doi.org/10.1016/j.ecolind.2014.04.049>.

Schou, J.C., Moeslund, B., Baastrup-Spohr, L., and Sand-Jensen, K. (2017). *Danmarks vandplanter (in Danish).* Klitmøller: BNFs Forlag.
