# Supplementary material for: The Dangers of Being a Small, Oligotrophic and Light Demanding Freshwater Plant across a Spatial and Historical Eutrophication Gradient in Southern Scandinavia
Source: Front Plant Sci. 2018 Feb 2;9:66. doi: 10.3389/fpls.2018.00066 (PMC5801560; doi:10.3389/fpls.2018.00066)
Supplement: Supplementary file 2 [file Data_Sheet_1.docx]

Supplementary Material

**The dangers of being a small, oligotrophic and light demanding freshwater plant across a spatial and historical eutrophication gradient in Southern Scandinavia**

Kaj Sand-Jensen, Hans Henrik Bruun, Tora Finderup Nielsen, Ditte Marie Christiansen, Per Hartvig, Jens Christian Schou and Lars Baastrup-Spohr

***Correspondence:**

Professor Kaj Sand-Jensen

ksandjensen@bio.ku.dk

# Supplementary Information

**Handling taxonomic differences between surveys**

For some taxa, the applied taxonomic level varied between surveys. In the field, identification to the species level is often possible, even on material insufficient for identification at the subspecific level. However, the different surveys may have made somewhat different choices as to which taxa to survey for and to spend later expert identification effort on. We handled such differences for the most problematic taxa as follows:

***Ranunculus aquatilis* (including *var. aquatilis* and *var. diffusus*)**

The varieties of *R. aquatilis* have not been consistently identified in any of the included surveys. In the final analyses, we included one taxa only, i.e. the species *R. aquatilis* sensu lato.

- In data from Denmark and Scania, all records of *R. aquatilis* and the two varieties (*var. aquatilis* and *var. diffusus*) were merged to one taxon (*R. aquatilis*) prior to data analysis.
- In Blekinge, all specimens were recorded as *R. aquatilis.*
- In Småland, plants were recorded as *R. aquatilis* or the two varieties (*var. aquatilis* and *var. diffusus*). However, *R. aquatilis was* recorded far more often than the varieties (30% vs 10% and 2.4%). Because of potential, but unknown, overlap (records at the species level and the variety level may or may not be assigned to the same grid cell), we chose to use only the records of *R. aquatilis* sensu lato in order not to artificially boost records of this species.

***Zannichellia palustris* (including *var. palustris* = *var. repens* and *var. pedicellata*)**

Different perceptions of species circumscription in the genus *Zannichelia* have prevailed in Scandinavia. We adopt the view of Schou et al. (2017) with *Z. palustris* as the only species, with other taxa as varieties*.* We included records of *Z. palustris* sensu lato only.

- In data from Denmark and Scania, all records of *Z. palustris* and the two varieties (*var. palustris* and *var. pedicellata*) were merged to one taxon (*Z. palustris*) prior to data analysis.
- In data from Småland, there were records of *Z. palustris* (5.5%) and *Z. palustris/major* (6.9%). We used the higher record (6.9%), in order not to underestimate the occupancy *Z. palustris,* as several occurrences of *Z. palustris* must be included in the *Z. palustris/major* records.
- In Blekinge, only the taxon *Z. palustris/major* was recorded and was scored as *Z. palustris* in our analyses.

***Utricularia australis/vulgaris***

*Utricularia* spp. are often found without flowers. The two species *Utricularia australis* and *Utricularia vulgaris* are extremely hard to discern without flowers, however. They have therefore been recorded in several of the surveys as *U. australis, U.* *vulgaris* and *U. australis/vulgaris*. In the final analyses we used the combined taxon *U. australis/ vulgaris.*

- In data from Denmark and Scania, all records of *U. australis* and *U.* *vulgaris* were merged into one taxon (*U. australis/vulgaris*) prior to data analysis.
- In Småland, plants were record as *U. australis, U.* *vulgaris* or *U. australis/vulgaris.* However, *U. australis/vulgaris* was recorded far more often than the other taxa (46% vs 6.6% and 4.1%, respectively). Because of potential overlaps (*U. australis/vulgaris* and *U. australis* or *U.* *vulgaris* may be recorded from identical grid cells), we chose to use records of *U. australis/vulgaris* only, in order not to artificially boost records of this taxa.
- In Blekinge, plants were record as *U. australis, U.* *vulgaris* or *U. australis/vulgaris.* However, *U. australis/vulgaris* was recorded far more often than the other taxa (38.6% vs 10.8% and 5.1%, respectively). Only records of *U. australis/vulgaris* were used for the reason mentioned above.

***Utricularia ochroleuca/stygia***

The two species *Utricularia ochroleuca* and *Utricularia stygia* are extremely difficult to differentiate when vegetative only. Thus, they have been recorded in several of the surveys as *U. ochroleuca, U.* *stygia* and *U. ochroleuca/stygia*. In the final analyses we used the combined taxon *U.* *ochroleuca/stygia.*

- In data from Denmark and Scania, all records of *U. ochroleuca* and *U.* *stygia* were merged into one taxon (*U.* *ochroleuca/Stygia*) prior to data analysis.
- In Småland, plants were record as *U. ochroleuca, U.* *stygia* and *U. ochroleuca/stygia.* However, *U. australis/vulgaris* was recorded more often than the other taxa (15% vs 6.2% and 7.3%). Because of potential overlaps (*U. ochroleuca/stygia* and *U. ochroleuca* or *U.* *stygia* may be recorded from identical grid cells), we chose to use records of *U. ochroleuca/stygia* only, in order not to artificially boost records of this taxon.
- In Blekinge, only *U. ochroleuca* (11.9) and *U.* *stygia* (12.5%) were recorded. Because of the overlap in habitat affinity between the two species (acid moors) we used maximum occurrence (12.5%) for the merged taxon in the further analysis.

**References**

Schou, J.C., Moeslund, B., Baastrup-Spohr, L., and Sand-Jensen, K. (2017). *Danmarks vandplanter (in Danish).* Klitmøller: BNFs Forlag.
